# Sustainable cultivation of the white truffle (*Tuber magnatum*) requires ecological understanding

**DOI:** 10.1007/s00572-023-01120-w

**Published:** 2023-07-18

**Authors:** Tomáš Čejka, Miroslav Trnka, Ulf Büntgen

**Affiliations:** 1 Department of Climate Change Impacts On Agroecosystems, Global Change Research Institute of the Czech Academy of Sciences, Bělidla 986/4, 603 00 Brno, Czech Republic; 2 Department of Ecology and Environmental Sciences, Faculty of Science, Palacký University Olomouc, Šlechtitelů 27, 783 71 Olomouc, Czech Republic; 3 Department of Agrosystems and Bioclimatology, Faculty of Agronomy, Mendel University, Zemědělská 1, 613 00 Brno, Czech Republic; 4 Department of Geography, Faculty of Science, Masaryk University, Kotlářská 2, 602 00 Brno, Czech Republic; 5 Swiss Federal Institute for Forest, Snow and Landscape Research WSL, Zürcherstrasse 111, 8903 Birmensdorf, Switzerland; 6 Department of Geography, University of Cambridge, Downing Place, Cambridge, CB2 3EN UK

**Keywords:** Ectomycorrhiza, Environmental change, Fungi, Global warming, Non-woody forest products, Truffle cultivation

## Abstract

The white truffle (*Tuber magnatum* Picco.; WT) is the most expensive and arguably also the most delicious species within the genus *Tuber*. Due to its hidden belowground life cycle, complex host symbiosis, and yet unknown distribution, cultivation of the enigmatic species has only recently been achieved at some plantations in France. A sustainable production of WTs under future climate change, however, requires a better ecological understanding of the species’ natural occurrence. Here, we combine information from truffle hunters with a literature review to assess the climatic, edaphic, geographic, and symbiotic characteristics of 231 reported WT sites in southeast Europe. Our meta-study shows that 75% of the WT sites are located outside the species’ most famous harvest region, the Piedmont in northern Italy. Spanning a wide geographic range from ~ 37° N in Sicily to ~ 47° N in Hungary, and elevations between sea level in the north and 1000 m asl in the south, all WT sites are characterised by mean winter temperatures > 0.4 °C and summer precipitation totals of ~ 50 mm. Often formed during past flood or landslide events, current soil conditions of the WT sites exhibit pH levels between 6.4 and 8.7, high macroporosity, and a cation exchange capacity of ~ 17 meq/100 g. At least 26 potential host species from 12 genera were reported at the WT sites, with *Populus alba* and *Quercus cerris* accounting for 23.5% of all plant species. We expect our findings to contribute to a sustainable WT industry under changing environmental and economic conditions.

**Supplementary Information:**

The online version contains supplementary material available at 10.1007/s00572-023-01120-w.

## Introduction

Truffles (*Tuber* spp.) are ectomycorrhizal fungi that grow in symbiotic relationships with the fine roots of their perennial host plants and form underground fruitbodies (Trappe and Claridge 2009). More than 200 truffle species have so far been described worldwide (Reyna and Garcia-Barreda 2014; Berch and Bonito 2016; Wan et al. 2017; Eberhart et al. 2020), of which about ten species are commonly harvested for human consumption (Hall et al. 2003). Of these, only three truffle species—the Burgundy (*Tuber aestivum* Vittad.), Périgord (*Tuber melanosporum* Vittad.), and White (*Tuber magnatum* Picco.; WT) truffle—receive great economic value (Stobbe et al. 2012; Reyna and Garcia-Barreda 2014; Pieroni 2016; Vita et al. 2018). Priced between tens and lower hundreds of Euros per kilogramme (Stobbe et al. 2012), the Burgundy truffle does not require high soil alkalinity and has a temperature range almost 2.5 times wider than that of the Périgord truffle (Čejka et al. 2020). The Périgord truffle prefers well-drained soils with a high pH level ~ 8 and grows in regions where temperature average reaches ~ 20 °C during hot summers and 4 °C during mild winters (Čejka et al. 2020). In addition to southern Europe, the Périgord truffle is cultivated in California, Chile, South Africa, and Australasia (Reyna and Garcia-Barreda, 2014; Thomas et al., 2019; Čejka et al. 2022), where each kilogramme can bring hundreds of Euros to the local economy (Oliach et al. 2021). The WT is with thousands of Euros/kg the most expensive *Tuber* species (Büntgen et al. 2019a). The price per kilogramme of fresh specimens can reach up to ~ 7000 Euros (Riccioni et al. 2016), and in Italy alone, the estimated total income from WTs exceeds 400 million Euros per year (Pieroni 2016). On a European scale, the production of WTs is valued at 900 million Euros (Lovrić et al., 2020). Its fame is due to an unmet demand because of short harvesting seasons from September to December, which mostly exists only at natural sites (Iotti et al. 2018), and still challenging cultivation. The mycelium of WTs not only prefers habitats in the last stage of plant succession but may also change its distribution rapidly between seasons compared to Burgundy and Périgord truffles (Iotti et al. 2014). While it is evidenced that mycelium is widespread in the soil (Leonardi et al. 2021; Iotti et al. 2014; Zampieri et al. 2010), ectomycorrhizae are few (Murat et al. 2005; Bertini et al. 2006; Leonardi et al. 2013; 2021). This, together with an insufficient understanding of the fungus’ life cycle (Paolocci et al. 2006), challenges the successful inoculation of host plants (Iotti et al. 2018), and sporadic cultivation of the species has only recently been achieved (Bach et al. 2021). In 2019 (2020), three (four) WT ascomycetes were successfully cultivated in at least one orchard in France under specific conditions (Bach et al. 2021). A sustainable production of WTs under future climate change, however, requires a better ecological understanding of the species’ natural occurrence.

Here, we combine unique insights from truffle hunters with information from 70 scientific publications to assess the climatic, edaphic, geographic, and symbiotic characteristics of 231 disjunct WT sites in southern and south-eastern Europe. The newly compiled ecological evidence is used to define the biotic and abiotic factors responsible for WT growth, to understand the socio-economic implications of an expected shift from WT hunting to WT cultivation, and to propose new research avenues to support the sustainability of traditional WT market models.

## Data and methods

To understand the ecological requirements of the WT growth, we conducted a literature review between 1 March and 15 May 2021 and 6 December 2022 to 15 February 2023 (Table S1 and Supplementary Data). We searched the Web of Science (Core Collection) and Scopus databases for peer-reviewed publications documenting the occurrence of WTs. The search was restricted to English language articles with publication dates after 1990 using a set of predefined search terms (Table S2). To confirm the accuracy of the results, we performed the search again, individually for all fungal and interdisciplinary, peer-reviewed, journals (Table S1 and Supplementary Data). In addition to the so-called ‘engine’ queries, we manually searched, translated, and reviewed articles and conference reports written in the local languages. This is because a substantial amount of information about the ecological requirements of the WTs has been published in non-indexed journals (Table S2).

In order to be considered in our review, the original publication had to introduce WT fruitbodies or describe the site ecology of WTs based on their confirmed occurrence. In addition to lists of protected fungal species that included the WT, we excluded publications analysing truffle products and those containing limited geographical descriptions. Similarly, publications with little data on ecological requirements as well as publications based on maps of potential distribution were excluded. To obtain unbiased ecological information from sites where WTs grow naturally, we excluded Bach et al. (2021), but compared their findings with our review in the discussion. We also excluded those publications that were located in areas where WTs grow and subsequent production at established plantation was the result of independent colonisation rather than targeted inoculation. In subsequent processing, we divided all publications based on fruitbody occurrence into four categories: (a) ‘genetics and polymerase chain reaction’, (b) ‘(nitrogen-fixing) bacteria’, (c) ‘biochemistry (volatile organic compounds, isotopic and elemental composition)’, and (d) ‘ecology’ (Supplementary Data).

In our literature review, we also focused on mean temperature and total precipitation (annual and seasonal), elevation, host species, and biogeochemical characteristics of suitable soils (Supplementary Data). Because each study was based on a different number of fruitbodies, often from multiple sites and even regions, we defined one site as representative of a single line of record in our review (Supplementary Data). Although some site records may have been subject of multiple studies, we did not correct for duplicates due to a lack of certainty. Where appropriate, approximate estimates of missing site coordinates were derived from the online gazetteer using the name of the location (https://www.gps-coordinates.net). In publications without site elevation information, we extracted this information using coordinates from the online OpenDataPortal1 composite elevation model with a horizontal resolution of one arcsecond (https://www.gpsvisualizer.com/elevation). Moreover, site coordinates were used to extract climate information from daily E-OBS gridded meteorological data for Europe with a resolution of 0.25° (10.24381/cds.151d3ec6) using Climate Data Operator software (Schulzweida 2021; function ‘remapnn’). Daily values were then averaged for seasons and years over the period 1991–2020.

## Results and discussion

### Bioclimatic conditions

**Fig. 1:**
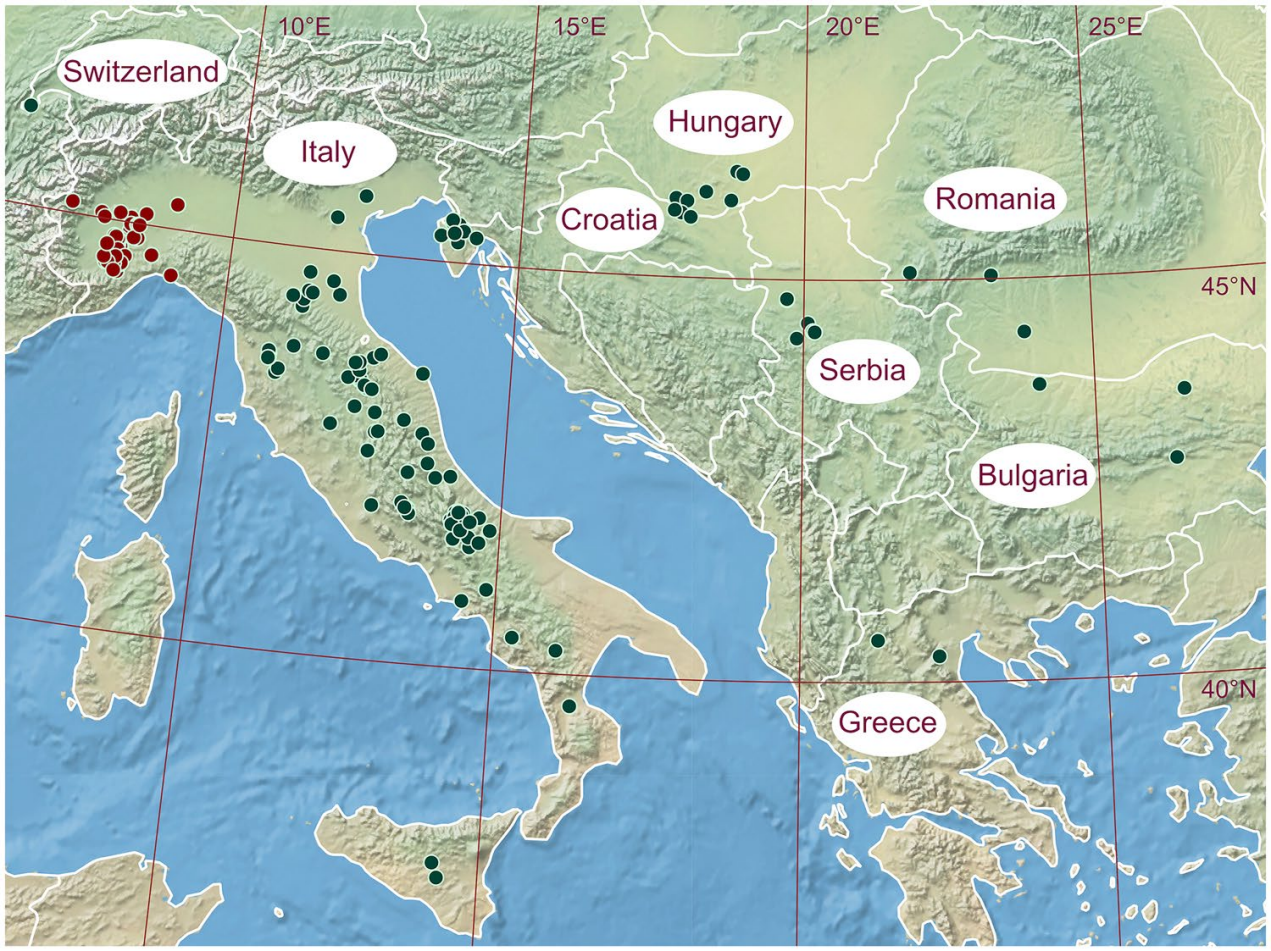
Distribution of 231 white truffle sites (green points), ~ 25% of which are in traditional harvesting region of Piedmont, Italy (dark red points). Only sites where fruitbodies have been confirmed in the literature are included (see Data and Methods for the explanation of potential duplicity and Tables S1 and S2 for reviewed publications). One site was also reported from Thailand (Suwannarach et al. 2017) but is not depicted in the figure

**Fig. 2:**
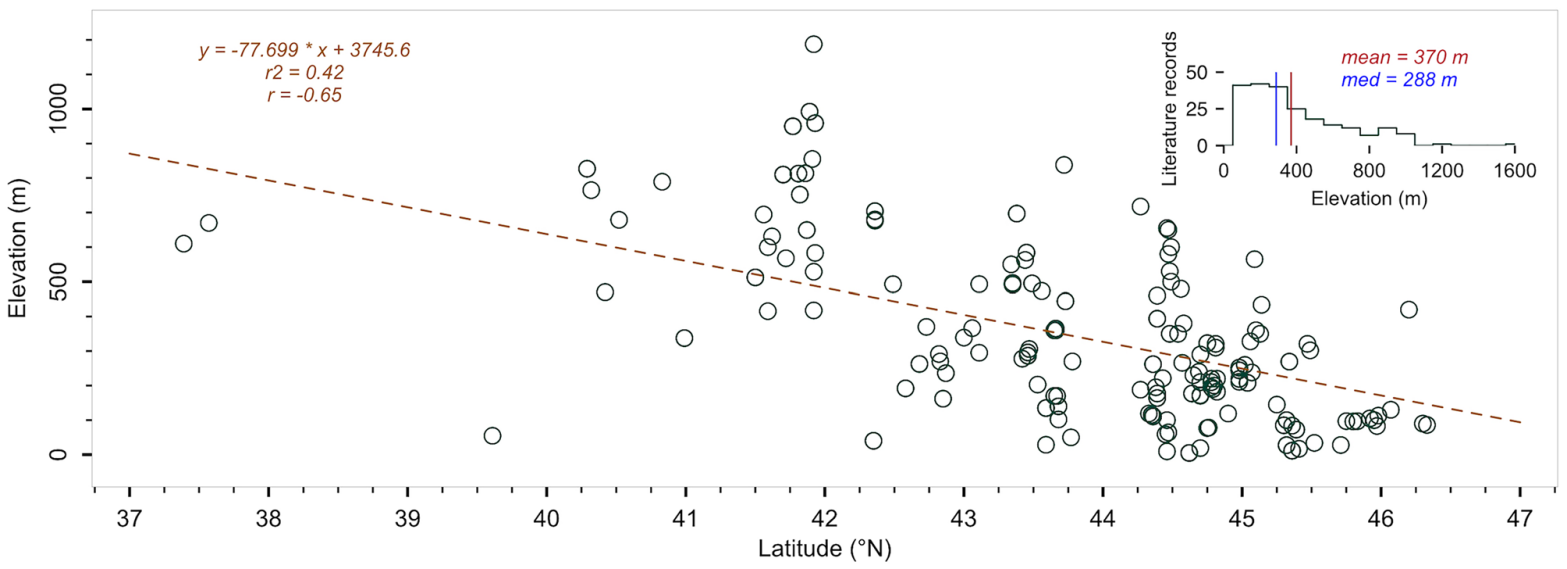
Relationship between elevation (*m*) and latitude (°N) based on literature data (dark green circles). The brown dashed line is a linear trend (*r*^2^ = 0.42). The histogram in the upper right corner shows the mean and median elevation of white truffle occurrence (370 and 288 m above sea level). One record from Thailand (Suwannarach et al. 2017) was included in the regression model but is not depicted in the figure

Contrary to the common belief that the WT is endemic to northern Italy (Pieroni 2016), the species occurs in natural habitats roughly between western Switzerland and eastern Bulgaria (~ 6°–27° E) and between Sicily and Hungary (~ 37.4–46.5° N) (Figs. 1 and 2; Büntgen et al. 2019a, b; Zambonelli et al. 2016; Vasquez et al. 2014). Of the data surveyed, only 25% of WT sites are reported from the Piedmont region in northern Italy. Another 52% of WT sites are scattered throughout the rest of the Apennine Peninsula, with a further 22% in Serbia (11 sites), Croatia (16), Hungary (8), Romania and Bulgaria (4 and 4), Slovenia (3), Greece (2), and Switzerland (1) (Fig. 1). Moreover, WT has been evidenced to grow rarely far from Europe at one site in northern Thailand at ~ 18° N, which is probably the southernmost limit of its global distribution (Suwannarach et al. 2017). However, the proportions and absolute values are possibly biased, primarily, because the 231 sites reported in Fig. 1 correspond to 78% of the literature review that we were able to compile, and the remaining 22% lack any geographic characteristics; second, because one site may be part of several studies, which is likely in areas with a higher site (publication) density, such as Italy. Therefore, there are probably fewer sites in Italy (at least those reported) with the remaining proportion to be likely higher in the Balkans/Pannonia. There is no published evidence on the distribution of WTs in many Balkan countries, including Bosnia and Hercegovina, Montenegro, Kosovo, Albania, and Macedonia. However, the absence of evidence does not imply the absence of WT existence and we believe that the former Yugoslavia must have been explored by truffle hunters. Given the generally suitable climate and similar vegetation composition to existing WT sites (see ‘Host plant and vegetation’), this raises interesting scientific and cultivation potential close to the species’ native range.

Evolutionary analyses of genetic structures (Frizzi et al. 2001), together with geographically specific ratios of climate-related stable isotopes, such as δ^15^N and δ^18^O (Krauß and Vetter 2020), of WTs help their market traceability from different regions (Bontempo et al. 2020). This proved useful not only for protection against illegal trade and for preserving the volatile (enzyme) spectrum under different storage conditions but also for reconstructing dispersal gradients/pathways (Buzzini et al. 2005; Pennazza et al. 2013; Zampieri et al. 2014; Vita et al. 2015; Segelke et al. 2020). Although still uncertain, the colonisation of new habitats may have been ongoing since the late Neogene, when the Apulo-Dalmatic Realm was formed (De Giuli et al. 1987). This caused the exposure and southward shift of the Adriatic Sea floor to 40–42°N and probably facilitated longitudinal migration of mycophagous species from Italy to the east (or vice versa from the Balkans). After the Last Glacial Maximum, the WT expanded from the central Italian and possibly the Balkan refugia, creating four distinct phylogeographic regions that differ in abundance and genetic diversity based on estimates of allelic richness and SSR markers (Rubini et al. 2005; Belfiori et al. 2020). This currently encompasses northern, northcentral, and southern Italy, as well as the Balkans/Pannonia (Belfiori et al. 2020). The traditional view accepts that WTs colonise new distant habitats through seasonal migration of mammals, such as wild boars (Piattoni et al. 2012), and this is likely the case for genetically distinct populations in northern and northcentral Italy (Belfiori et al. 2020). However, we suggest that birds may also play a role. Avifauna act as the dominant vector for the dispersal of viable truffle spores over distances of up to ~ 1500 km (Caiafa et al. 2021). Avian transport may explain the similarity in genetic diversity between the southern Italian and Greek WT sites (Belfiori et al. 2020), which are separated by a strait between the southern Adriatic and the northern Ionian Sea.

**Fig. 3:**
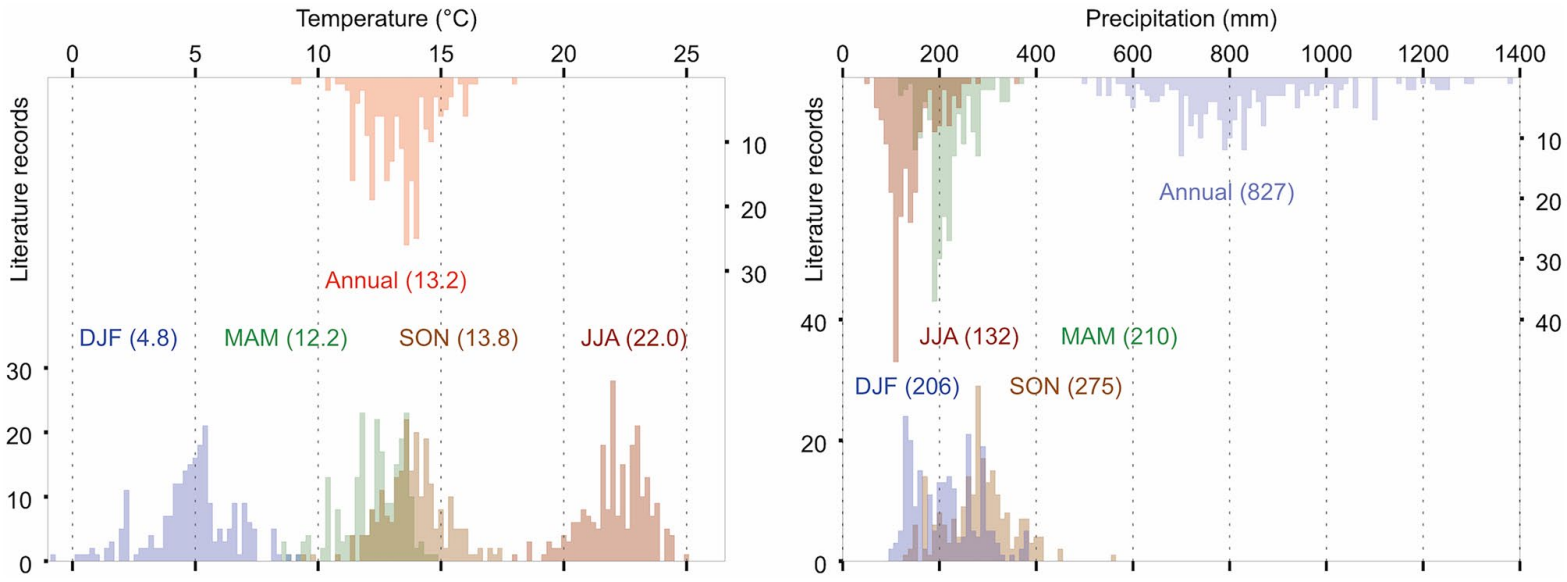
Climatic conditions of white truffle sites expressed as annual and seasonal temperature means (left) and precipitation totals (right), derived from the literature review

In addition to migration routes associated with evolutionary origins, the distribution of WTs is governed by many factors, including climate and possibly its seasonality (i.e., the amplitude between summer and winter). In the west and northwest, their natural distribution is likely limited by the humid temperate (oceanic) climate, which is cooler and less distinct in seasonality compared to the Mediterranean climate (Beck et al. 2018) where the species mostly thrives. The continental climate, which may limit the WT distribution in the east, has a pronounced annual seasonality with warmer summers but winter temperatures below freezing rather than above (Beck et al. 2018). The mean winter isotherm of 0 °C appears to limit the species’ distribution up north, because fruitbodies need at least 0.4 °C (1st percentile) during this period for their formation (Fig. 3). Since frosts are more common north of the fungus’ native range and are destructive to other truffle species to varying degrees depending on their water-holding capacity (Zampieri et al. 2011), emerging cultivation of WT would benefit from better understanding of fungus’ short- and long-term tolerance to sub-zero temperatures. Moreover, WTs may grow in specific microclimatic conditions outside their acceptable climatic range (defined in Figs. 1 and 3), for example, in the Apennine Peninsula, where suitable habitats are associated with high terrain ruggedness (Bragato et al. 1992). Difficult access to these and other sites, for example, in Croatia, is another reason why we consider Fig. 1 to be an underestimate for sites in the Balkans.

In terms of elevation, the significant relationship shows that the more northerly the sites are, the lower they are to sea level (*r*^2^ = 0.42, *p* < 0.01), suggesting similar climatic conditions for WT growth. These are mainly determined by temperature (Miyamoto et al. 2018) related to the elevation of the sites, which ranges from < 100 m asl in Serbia and southern Hungary (Marjanović et al. 2010; Büntgen et al. 2019a) to ~ 1000 m asl in central Italy (Iotti et al. 2012; 2014; Leonardi et al. 2013; Lalli et al. 2015). Note, however, that Fig. 2 is likely biased because some publications did not include latitude and altitude variables. Despite this broad elevation profile, WTs grow on average at lower elevation (~ 370 m asl; Fig. 2) as compared to the Burgundy and Périgord truffles, whose optimal elevations are around ~ 570 and ~ 620 m asl, respectively (Čejka et al. 2020). This is due to the fact that their annual temperature optima are 1.4–3.1 °C lower than those of the WT, which thrives best at sites with ~ 13 °C per year (1st–99th percentile equals to 10.3–16.0 °C).

Annual temperature range translates into an average of ~ 12, 22, 14, and 5 °C for Mar–May, Jun–Aug, Sep–Nov, and Dec–Feb, respectively (Fig. 3). The warmest mean air temperature for WT growth in Jun–Aug is 24.3 °C (99th percentile), which is about four degrees above the physiological optimum for mycelial development in soil (Iotti et al. 2018). Excessive temperatures then reduce the amount of mycelium in the topsoil (~ 10 cm) (Marjanović et al. 2015), which is likely why the WT develops extra-radical mycelium in horizons below 30 cm (Iotti et al. 2018; Le Tacon 2016; Ceruti et al. 2003) where the soil profile is deep enough. This is in contrast to the fruitbodies of Burgundy and Périgord truffles, which grow closer to the surface because they are better adapted to temperature extremes (Büntgen et al. 2021; Mello et al. 2006) and on average grow in shallower soils. Although mycelial abundance decreases under drought-induced stress (Iotti et al. 2014; 2018), Burgundy and Périgord fruitbodies (Büntgen et al. 2019c) also better tolerate short-term precipitation deficits in summer, despite having similar requirements as the WT (~ 135–160 mm; Čejka et al. 2020). This species, however, does not have a well-developed peridium that otherwise protects against water transpiration through mechanical, metabolic, and biochemical processes (Monaco et al. 2021; Zarivi et al. 2000). This will put pressure on existing and upcoming irrigation systems as the water requirements of the WT are likely to be higher than those of traditionally cultivated truffle species. In contrast, at sites north of the Mediterranean, including Geneva, Switzerland, WTs tolerate summer excess precipitation up to 180% of normal (98th percentile) (Büntgen et al. 2019b). Expected precipitation increase and projected warming are likely to shift the northernmost limit of the WT and shift its habitat to central and western Europe. In the future, emerging migration routes and newly colonised territories will improve our understanding of the forming soil conditions required for WT growth.

## Edaphic variation

**Fig. 4:**
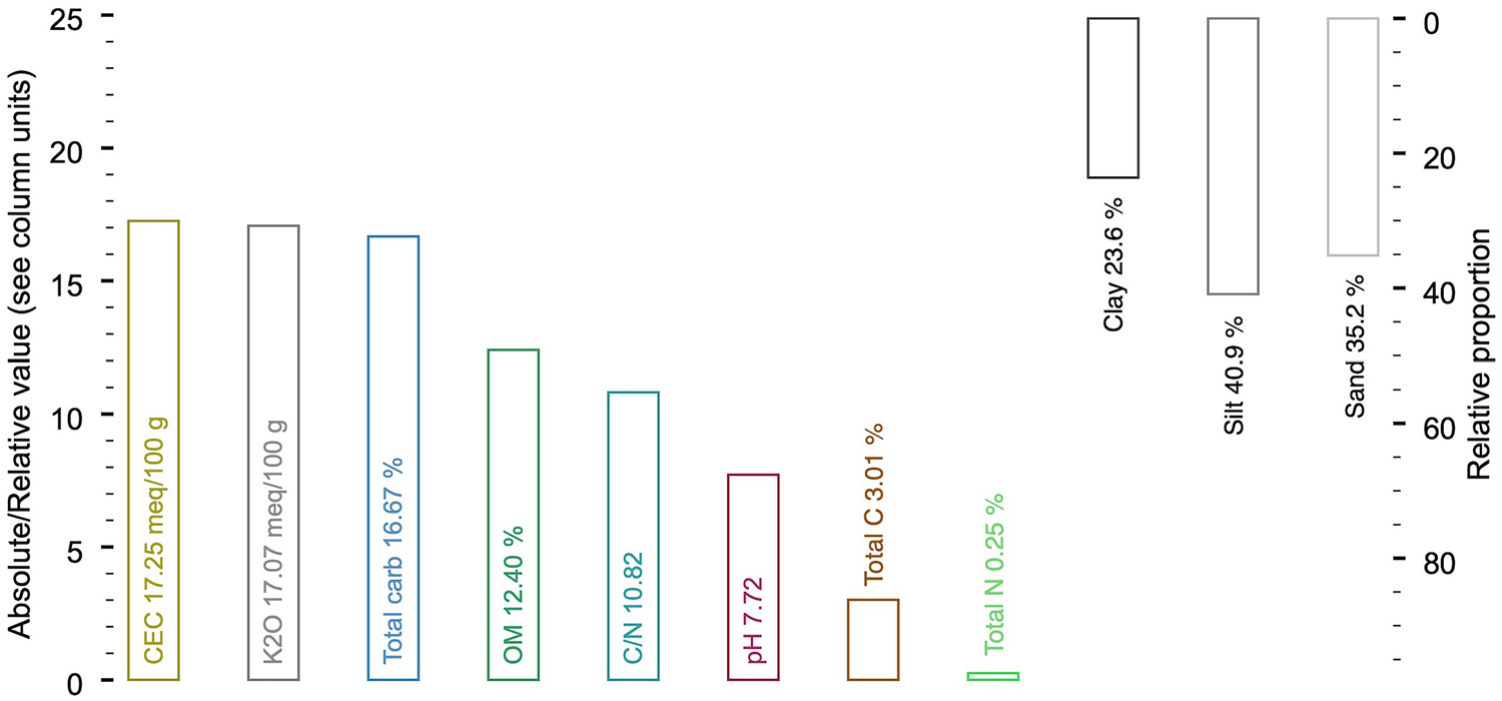
Geochemical conditions of white truffle soils. CEC, cation exchange capacity; total carb, total carbonates; OM, organic matter content; Total C, total carbon; Total N, total nitrogen

Our literature review shows that soils where WTs grow have an average pH level of ~ 7.7 (Fig. 4). However, pH at WT sites ranges from neutral to alkaline (Marjanović et al. 2015), unlike Périgord truffles, which are restricted to alkaline environments (Čejka et al. 2020). The non-limiting nature of soil acidity/alkalinity for WT growth is likely to allow growers to cultivate this fungus’ over a larger area outside its native range if the following soil conditions are met. While Périgord truffles also require well-drained soils with higher sand/silt content, WT soils contain more clay (> 20%), especially in the Balkans and Pannonia (Fig. 4; Bragato et al. 2004; 2010). Therefore, more evenly distributed grain fractions allow both higher cation exchange capacity (~ 17 meq/kg; Fig. 4) and higher soil macroporosity, i.e., > 15% proportion of pores > 50 μm in size (Bragato et al. 2010). The latter, together with the elongation and pore size of soil aggregates, seems to determine the presence or absence of WTs in the Apennines (Bragato et al. 2010) and possibly also in Istria. Moreover, at these WT sites, the silt content dominates (45%) at the expense of clay (< 20%) (Supplementary Data). In natural sites, these specific conditions are related to the vertical redistribution of mineral and organic matter during initial soil formation due to flooding, especially in the Balkans and Pannonia and in combination with landslide processes also in Italy and Istria (Favre et al. 2002). Local sediments at the latter WT sites also typically exhibit considerable amounts of carbonates (15%) (Bragato et al. 2010), which is consistent with our literature mean (~ 18%; Supplementary Data). In contrast, Hungarian and Balkan WT sites show much lower abundances of around 10%, which may be influenced by the lack of upstream resources of carbonates and/or the low-energy sediment flux that is typical for south-eastern European lowlands (Słowik et al. 2020). Although nitrogen content is relatively low (0.19–0.26%), the organic matter content in Italy is three times higher (~ 14%) than that of WT sites in the Balkans (4.5%) (Supplementary Data). Together with a *C*/*N* ratio of around 7 at Italian sites, this might be ascribed to relatively slow decomposition rates (Fig. 4). The higher ratio of this parameter in the Hungarian and Balkan lowlands, which are exposed to quasi-periodic flooding, corresponds to faster decomposition rates associated with elevated microbial activity in the uppermost soil layer (Büntgen et al. 2019a). Our review highlights large variance in soil abiotic factors, and we argue that new research should include the biotic dimension, specifically microbial and fungal communities, not only to understand occurrence but also in relation to the spatial exchange, vertical supply, and seasonal dynamics of nutrients (Iotti et al. 2018).

It is generally accepted that microbes play an important role in the growth of WTs (Pavić et al. 2013). However, the attribution of specific bacterial genera/species to a particular process, including fruitbody ripening, aroma production, and nutritional enhancement, is uncertain in the noisy natural background (Niimi et al. 2021; Splivallo et al. 2011; Pavić et al. 2013). Unravelling the links is challenged by the fact that bacterial composition is independent of the maturation stage and varies between regions (Niimi et al. 2021). Therefore, the implementation of different soil conditions and different bacteria (and fungi) to support the growth of other high-prized truffle species (Monaco et al. 2020; Piñuela et al. 2020) in WT plantations would complement our current, albeit still descriptive, knowledge. So far, the dominant bacterial genera include (but are not limited to) *Sinorhizobium*, *Rhizobium*, *Bradyrhizobium*, and *Pseudomonas* (Barbieri et al. 2007), which mainly fix nitrogen from the atmosphere into the soil (TN 0.24%; Fig. 4; Barbieri et al. 2010), where it becomes available to coexisting saprotrophic and ectomycorrhizal fungi (Corrales et al. 2017; Marozzi et al. 2023). Saprotrophs associated with WT growth include the common genera *Mortierella* and *Fusarium* (Mello et al. 2010) and the plant pathogens *Peniophora*, *Paecilomyces* and *Verticillium* (Pacioni et al. 2007). In terms of functional guilds, ectomycorrhizal fungi dominate WT habitats, including species from the *Thelephoraceae* and *Sebacinaceae* families (Marjanović et al. 2020). However, despite the considerable overlap in ectomycorrhizal species composition between productive and unproductive sites (ecologically comparable to productive sites) (Leonardi et al. 2013), fungal species of the genus *Sebacina* appear to be associated only with WT growth (Leonardi et al. 2013; Murat et al. 2005). A recent comparison from Italy revealed that among bacteria phylum *Firmicutes* was more likely to be associated with the presence of WT, whereas several others including *Gemmatales* and *Nitrososphaerales* were associated with its absence (Sillo et al. 2022). In addition to microbial communities in productive soils, those present in the peridium may affect its thickness (Monaco et al. 2021) and possibly ripeness of the gleba, where bacterial populations determine fruitbody size (Sillo et al. 2022). As complexity (and unfortunately uncertainty) increases with mounting evidence, the truffle community has recently started calling for the identification of a ‘core truffle microbiome’, a subset of taxonomic individuals that is common across WT sites in space and time (regions and seasons) (Marozzi et al. 2023; Monaco et al. 2022; Sillo et al. 2022). A systematic approach, followed by attribution of these taxa to functional guilds, including ectomycorrhizal and saprotrophic groups, would allow a more contextualised comparison of site-specific microbial communities. This approach could reduce the uncertainty in comparing productive and unproductive sites, where selection of the latter is often based on biased criteria that rely on personal experience. Although associations between taxonomic presence and fruitbody occurrence remain purely descriptive and require the inclusion of reliable identification of WT, either by fungal-specific antibodies or PCR approaches (Lanfranco et al. 1993; Neuner-Plattner et al. 1999), their existence suggests the importance of beneficial fungal interactions that add complexity to already known associations with host plants (Hall et al. 2003; Moser et al. 2017).

## Host plant and vegetation

**Fig. 5:**
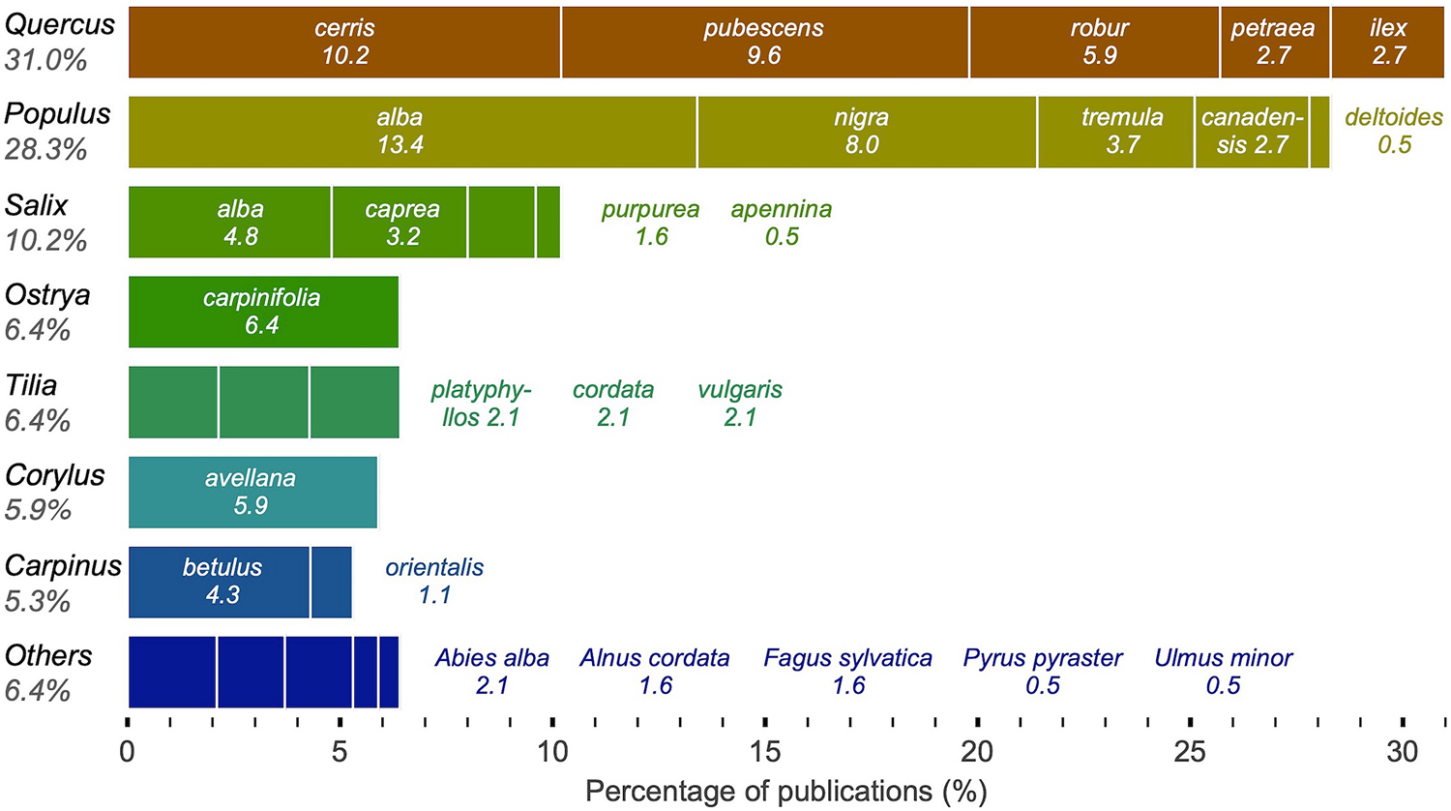
Most common host plants of white truffle expressed as percentage of publications in which the species was mentioned (horizontal axis). Percentages under genera on the left are cumulative for all species

WTs mainly grow in association with oaks (~ 30%) (Fig. 5), which include both Mediterranean (*Q. pubescens*, *Q. cerris*, and *Q. ilex*) and temperate species (*Q. robur* and *Q. petraea*). At sites outside Italy, host species other than oaks predominate, with nearly 30% of the literature reporting associations with poplars, including *P. nigra*, *P. tremula*, *P. canadensis*, *P. deltoides*, and *Populus alba*, the most common host species ever (~ 13%). Every tenth publication documents symbiosis with willows, also listing four species (*Salix caprea*, *S. alba*, *S. purpurea*, and *S. apennina*). The remaining host plants with a relative abundance < 10% are represented by Hop hornbeam (*Ostrya carpinifolia*), hornbeam (*Carpinus betulus* and *C. orientalis*), linden (*Tilia platyphyllos*, *T. cordata*, and *T. vulgaris*), and hazel (*Corylus avellana*). The last few percent of all known host plants is represented by five species, all from different genera: *Abies alba*, *Alnus cordata*, *Fagus sylvatica*, *Pyrus pyraster*, and *Ulmus minor* (for the host species listed see Supplementary Data and Caramiello 1968; Montacchini 1968; Montacchini and Caramielo 1968; Lulli et al. 1991; Panini et al. 1991; Bragato et al. 1992; Lulli et al. 1992; Mirabella et al. 1992; Lorenzelli et al. 1996; Hall et al. 1998; Rubini et al. 2001; Mello et al. 2005; Bertini et al. 2006; Bragato et al. 2010; Ciaschetti et al. 2010; Di Massimo et al. 2010; Donnini et al. 2010; Gregori et al. 2010; Pavarino et al. 2010; Iotti et al. 2012; Piattoni et al. 2012; Christopoulos et al. 2013; Leonardi et al. 2013; Iotti et al. 2014; Salerni et al. 2014; Lalli et al. 2015; Murat et al. 2018; Vita et al. 2018; Büntgen et al. 2019a, b; Gregorčič et al. 2020; Petrella et al. 2020). Note, however, that the relative abundance of host plants is biased by those publications that have revealed their existence, and not all WT-host plant combinations have been confirmed by molecular identification of both partners in WT mycorrhizas. Moreover, since the information is related to the publication (not a site), we are unable to quantify regionally specific information on the occurrence of particular genera/species. Despite this limitation, however, the literature indicates that poplars (*Populus*) are typical of Italian WT sites, while sites on the other side of the Adriatic Sea are mostly represented by oaks (*Quercus*) (Montacchini, 1968).

Apart from the knowledge that host plants track their natural distribution, the existing literature lacks a deeper understanding of the role (e.g., performance) of these species in the formation and maturation of WTs. Moreover, different ages of forest stands, and thus different successional stages (ranging from meadow to old-growth forest), are associated with different production rates (Heinonen et al. 2021). Independent multi-year observations from floodplain habitats in southern Hungary (I. Bagi pers. comm.) suggest that only oak (*Quercus*) forest stands older than ~ 80–90 years are capable of producing WTs, compared to nearby, albeit younger, trees of the same species. On the other hand, WTs from a plantation in southern France were harvested no more than 15 years after planting its *Quercus* host (Bach et al. 2021). This discrepancy suggests two non-exclusive premises. First, natural habitats require a long time to establish suitable conditions for WT growth that can otherwise be mimicked in plantations in a much shorter period. Second, fruitbody formation is affected by other unknown factors because WTs occur only after several years in natural sites, despite the soil chemistry is comparable between reviewed and French sites (Supplementary Data and Table S1 in Bach et al. 2021). Therefore, we assume that the occurrence of WT depends on the abundance and diversity of succession stage-specific microbiota, which is related to a particular land-use (Graziosi et al. 2022; Heinonen et al. 2021). Our understanding would benefit from the inclusion of historical and silvicultural maps into WT research.

Different production rates could be related to the greater tolerance of WT to shading, as uncontrolled old native sites typically have denser canopies (Bragato et al. 2004). Burgundy truffles also have comparatively low sunlight requirements (Molinier et al. 2013) but prefer younger trees in the primary successional stage on non-native sites. Young, rather than old forest stands are favourable to Périgord truffle, which, unlike other two species require much more sunlight (Garcia-Barreda and Reyna 2013). Hence, to better understand the requirements for growing WTs, it is necessary to include all these and other common habitat characteristics such as canopy structure, extent of bare soil, horizontal/vertical distance from water, and Ellenberg and Shannon indices. Since multiple genera of trees and shrubs (e.g., *Crataegus*, *Cornus*, *Fraxinus*, *Aesculus*) are often found at WT sites (Bragato et al. 2004; Marjanović et al. 2010; Iotti et al. 2014; Popović-Djordjević et al. 2019), floristic composition beyond known hosts combined with indices of vegetation diversity is likely to become valuable habitat indicators for WT, as has been demonstrated for other truffle species (Moser et al. 2017).

## Conclusion and implications

Based on joint information from truffle hunters and a literature review consisting of 70 publications, we estimated climatic, edaphic, geographic, and symbiotic characteristics of WT growth from of 231 disjunct sites in southern and south-eastern Europe. Our meta-study showed that 75% of the reported WT sites are located outside the species’ most famous harvest region in Piedmont, northern Italy. Spanning a wide geographic range from ~ 37° N in Sicily to ~ 47° N in Hungary, and elevations between sea level in the north and 1000 m asl in the south, all WT sites are characterised by mean winter temperatures > 0.4 °C and summer precipitation totals of ~ 50 mm. Often formed during past flood or landslide events, current soil conditions of the WT sites exhibit pH levels between 6.4 and 8.7, high macroporosity, and a cation exchange capacity of ~ 17 meq/100 g. At least 26 potential host species from 12 genera were reported at the WT sites, with *Populus alba* and *Quercus cerris* accounting for 23.5% of all species.

We also used existing ecological information to promote new research directions that could not be addressed without the possibility to cultivate this iconic fungus. Many suitable habitats outside the species’ traditional harvest region in Piedmont in northern Italy, such as Albania or Kosovo, remain unexplored, and the significance of the 0 °C winter isotherm in WT formation is unknown. Starting cultivation could benefit from the species’ wide pH range (6.4–8.7), as well as attribute the role of growth-promoting bacterial species to a specific stage in fungus’ development. Likewise, descriptive understanding of host vegetation would benefit from an explanation of the role of forest age and successional stages in the formation of WTs.

Looking to the future, the destruction of WT habitat caused by anthropogenic climate change combined with the transition from hunting to cultivation is likely to introduce new economic challenges. In addition to already described negative effects of summer drought and the expansion of viticulture on yields and suitable habitats of WTs throughout the Mediterranean (Büntgen et al. 2019b; Pieroni 2016), regions with increasing temperatures that are located in humid continental climates (e.g., central Europe, the interior of the Balkan Peninsula), will be particularly vulnerable to precipitation extremes and subsequent flooding events (Alfieri et al. 2015; Stadtherr et al. 2016; Blöschl et al. 2020). Therefore, excessive waterlogging and widespread inundation in existing alluvial/riparian WT habitats would likely cease the abundance of mycorrhizae and production of fruitbodies, as has been observed for the Burgundy truffle elsewhere (Thomas, 2021). The consequent decline in WT production may be devastating, especially in economically poor regions such as southern Hungary, where so far stable harvests of WTs have added much economic value to otherwise struggling and neglected rural communities.

Should the production of cultivated truffles increase and become sustainable, the subsequent devaluation and price decline of fresh specimens due to increased supply could cause tensions from traditional hunters and possibly even the abandonment of the activity. A competitive and locally disconnected production, which runs counter to what we consider a sustainable and reciprocal truffle industry, is likely to loosen personal (and inter-company) ties and even slow scientific progress in the short-term. In the longer term, however, the advent of cultivation opens up a new testing ground, for many new and yet to come challenges in WT research, such as those discussed in this study.

### Supplementary Information

Below is the link to the electronic supplementary material.

Supplementary file1 (XLSX 195 KB)

Supplementary file2 (DOCX 64 KB)

## Data Availability

The data used in this article may be requested from the authors upon reasoned request.
