# Supplementary material for: Sustainable cultivation of the white truffle (Tuber magnatum) requires ecological understanding
Source: Mycorrhiza. 2023 Jul 18;33(5-6):291–302. doi: 10.1007/s00572-023-01120-w (PMC10752849; doi:10.1007/s00572-023-01120-w)
Supplement: Supplementary file 2 — Supplementary file2 (DOCX 64 KB) [file 572_2023_1120_MOESM2_ESM.docx]

**Supplementary Material**

**Sustainable cultivation of the white truffle (*Tuber magnatum*) requires ecological understanding**

Tomáš Čejka^1,2^*, Miroslav Trnka^1,3^, Ulf Büntgen^1,2,4,5^

*^1^Department of Climate Change Impacts on Agroecosystems, Global Change Research Institute of the Czech Academy of Sciences, Bělidla 986/4, 603 00 Brno, Czech Republic. ^2^Department of Geography, Faculty of Science, Masaryk University, Kotlářská 2, 602 00 Brno, Czech Republic. ^3^Department of Agrosystems and Bioclimatology, Faculty of Agronomy, Mendel University, Zemědělská 1, 613 00 Brno, Czech Republic. ^4^Swiss Federal Institute for Forest, Snow and Landscape Research WSL, Zürcherstrasse 111, 8903 Birmensdorf, Switzerland. ^5^Department of Geography, University of Cambridge, Downing Place CB2 3EN, Cambridge, United Kingdom.* **Corresponding author: tomas.cejka.94@gmail.com*

**Supplementary Table S1.** List of all reviewed journals from which 70 publications were retrieved (see Supplementary Data for all details regarding used data). Any potential information that could have been lost due to strict review criteria is included in the discussion.

| **Number** | **Journal** |
| --- | --- |
| 1 | 3 Biotech |
| 2 | Acta Mycologica |
| 3 | Annals of Microbiology |
| 4 | Applied Soil Ecology |
| 5 | Biology and Fertility of Soils |
| 6 | BMC Microbiology |
| 7 | Canadian Journal of Microbiology |
| 8 | Chemistry Biodiversity |
| 9 | Environmental Microbiology Reports |
| 10 | Environmental Microbiology |
| 11 | Environmental Pollution |
| 12 | FEMS Microbiology Ecology |
| 13 | FEMS Microbiology Letters |
| 14 | Food and Chemical Toxicology |
| 15 | Food Chemistry |
| 16 | Food Control |
| 17 | Food Microbiology |
| 18 | Food Research International |
| 19 | Frontiers in Microbiology |
| 20 | Fungal Biology |
| 21 | Fungal Biology Reviews |
| 22 | Fungal Ecology |
| 23 | Fungal Genetics and Biology |
| 24 | Journal of Chromatography A |
| 25 | Journal of Pharmaceutical and Biomedical Analysis |
| 26 | LWT |
| 27 | Microbial Ecology |
| 28 | Microbiological Research |
| 29 | Microorganisms |
| 30 | Mycobiology |
| 31 | Mycological Research |
| 32 | Mycologist |
| 33 | Mycoscience |
| 34 | New Phytologist |
| 35 | Phytochemistry |
| 36 | Phytotaxa |
| 37 | Plant and Soil |
| 38 | Plant Biosystems |
| 39 | PLOS One |
| 40 | Postharvest Biology and Technology |
| 41 | Scientific Reports |
| 42 | Soil Biology and Biochemistry |
| 43 | Sydowia |
| 44 | The Lancet |
| 45 | Transactions of the British Mycological Society |
| 46 | Trends in Food Science & Technology |
| 47 | Trends in Plant Science |
| 48 | Turkish Journal of Botany |

**Supplementary Table S2.** Search words. When searching using the search engine (Web of Science and Scopus), we only consider publications classified in the categories: ‘(research) article’, ‘review (article)’, ‘mini reviews’, ‘letter’, ‘early access’, ‘correspondence’, ‘discussion’, ‘short communication’, and ‘book review’, excluding ‘proceeding paper’, ‘news item’, ‘correction’, ‘meeting abstract’, ‘encyclopaedia’, ‘book chapters’, ‘conference info’, ‘editorials’, ‘news’, and ‘other’.

| ‘magnatum’ ‘soil’ OR ‘white truffle’ ‘soil’ |
| --- |
| ‘magnatum’ ‘climate’ OR ‘white truffle’ ‘climate’ |
| ‘magnatum’ ‘host’ OR ‘white truffle’ ‘host’ |
| ‘magnatum’ ‘precipitation’ OR ‘white truffle’ ‘precipitation’ |
| ‘magnatum’ ‘temperature’ OR ‘white truffle’ ‘temperature’ |
